# Microbial signatures of neonatal bacterial meningitis from multiple body sites

**DOI:** 10.3389/fcimb.2023.1169101

**Published:** 2023-08-22

**Authors:** Yuyang Hou, Meng Zhang, Qiannan Jiang, Yuping Yang, Jiang Liu, Ke Yuan, Zheng Sun, Xiuxiang Liu

**Affiliations:** ^1^ Women and Children’s Hospital, Qingdao University, Qingdao, Shandong, China; ^2^ Qingdao Medical College, Qingdao University, Qingdao, Shandong, China; ^3^ Key Laboratory of Dairy Biotechnology and Engineering, Ministry of Education, Inner Mongolia Agricultural University, Hohhot, Inner Mongolia, China; ^4^ Single-Cell Center, CAS Key Laboratory of Biofuels and Shandong Key Laboratory of Energy Genetics, Qingdao Institute of BioEnergy and Bioprocess Technology, Chinese Academy of Sciences, Qingdao, Shandong, China; ^5^ Qingdao OE Biotechnology Company Limited, Qingdao, Shandong, China

**Keywords:** 2bRAD-M sequencing, blood microbiome, skin microbiome, cerebrospinal fluid microbiome, neonatal meningitis

## Abstract

As a common central nervous system infection in newborns, neonatal bacterial meningitis (NBM) can seriously affect their health and growth. However, although metagenomic approaches are being applied in clinical diagnostic practice, there are some limitations for whole metagenome sequencing and amplicon sequencing in handling low microbial biomass samples. Through a newly developed ultra-sensitive metagenomic sequencing method named 2bRAD-M, we investigated the microbial signatures of central nervous system infections in neonates admitted to the neonatal intensive care unit. Particularly, we recruited a total of 23 neonates suspected of having NBM and collected their blood, cerebrospinal fluid, and skin samples for 2bRAD-M sequencing. Then we developed a novel decontamination method (Reads Level Decontamination, RLD) for 2bRAD-M by which we efficiently denoised the sequencing data and found some potential biomarkers that have significantly different relative abundance between 12 patients that were diagnosed as NBM and 11 Non-NBM based on their cerebrospinal fluid (CSF) examination results. Specifically, we discovered 11 and 8 potential biomarkers for NBM in blood and CSF separately and further identified 16 and 35 microbial species that highly correlated with the physiological indicators in blood and CSF. Our study not only provide microbiological evidence to aid in the diagnosis of NBM but also demonstrated the application of an ultra-sensitive metagenomic sequencing method in pathogenesis study.

## Introduction

Neonatal bacterial meningitis (NBM) is a common central nervous system infection that can seriously affect the health and growth of infants (Liu et al., 2020). Even though many interventions have been developed to treat NBM, including vaccinations against meningococcus, monoclonal antibodies, steroids, and antibiotics, it remains to be a devastating infection of the central nervous system among newborns (de Louvois et al., 2005; Liu et al., 2020), affecting 0.49 infants per 1000 live births, causing 15.9% deaths, and 21.6% neurological sequelae (Ben Hamouda et al., 2013).

The importance of early diagnosis and antimicrobial therapy for NBM infants cannot be overstated, but the challenge is still great (Bedetti et al., 2019; Liu et al., 2020). For example, NBM can be caused by a variety of causes and is characterized by atypical clinical manifestations. In neonates, it may only present with crying and feeding difficulties, rather than typical central nervous system infection, including, seizures or a bulging fontanelle. The absence of manifestations in neonates makes the diagnosis of NBM much more difficult than in older children and adults (Baud and Aujard, 2013). To address the above question, laboratory testing is followed by clinical manifestations based on the current standard for diagnosing infections (Wilson et al., 2019). Although a positive cerebrospinal fluid (CSF) culture is considered to be the gold standard, it requires time for viable organisms in the CSF to be detected on media (Graff et al., 2021) and easily affected by antibiotic usage (Srinivasan et al., 2012). Moreover, there are many limitations in other traditional diagnostic approaches, e.g., CSF parameters such as CSF glucose, white blood cell (WBC) count and protein are limited by their sensitivity to time (Smith et al., 2008; Rajesh et al., 2010); PCR is limited by accurate initial hypothesis (Westblade et al., 2016); and serologic testing of pathogen-specific antibodies suffer from uncertainty (Graff et al., 2021). All these limitations lead to a missing of accurately identified pathogens in approximately 50% meningoencephalitis cases (Granerod et al., 2010).

As advanced laboratory assays, metagenomic approaches (includes amplicon sequencing and whole metagenome sequencing, WMS) are now being used clinically to improve the diagnostic accuracy of pathogens and avoid inappropriate therapies (Miller et al., 2019; Zanella et al., 2019; Piantadosi et al., 2021; Guo et al., 2022). Compared with amplicon sequencing, WMS has more potential in clinical practice since amplicon sequencing suffers from off-target amplification (Bedarf et al., 2021), biased abundance estimation, limited taxonomic resolution, insensitivity to degraded DNA, and unable to simultaneously capture all microorganisms (e.g., bacteria, fungi, archaea, and virus) in one sequencing (Knight et al., 2018). However, WMS is not a perfect solution since it also suffers from high cost and low microbial biomass samples, while the limited availability and volume from invasive procedures such as lumbar puncture makes it difficult to extract high quality DNA from CSF (Graff et al., 2021; Morsli et al., 2022). To overcome the difficulties in metagenomic sequencing of low microbial biomass such as CSF and blood samples, we innovatively employed 2bRAD-M in this study, which has been proven to be effective in dealing with 1pg total DNA, 50 bp highly degraded DNA, and 99% host contaminated DAN samples (Sun et al., 2022a).

In previous studies, little effort has been made to study the microbial signatures of CSF in NBM patients, and systematic sampling (environment, skin, and blood) has rarely been conducted. By recruiting 23 patients suspected of having NBM (**Methods**) from the neonatal intensive care unit (NICU) and leveraging their microbial signatures from multiple body sites decoded by the cutting-edge 2bRAD-M sequencing, our study aims to identify the underlying associative agents and risk factors associated with central nervous system (CNS) infections in neonates, as well as to provide a basis for the early diagnosis and treatment of neonatal bacterial meningitis in the NICU.

## Methods

### Sampling

The diagnosis of neonatal bacterial meningitis is difficult due to the inconspicuous clinical manifestations, thus the diagnosis mainly depends on the vigilance of the disease. We recruited children with suspected central nervous system (CNS) infectious diseases using the following criteria: 1. pathogenic bacteria detected in blood cultures; 2. clinical and laboratory tests both suggest Central nervous system infection; 3. the child did not improve after receiving antibiotic therapy. For the 23 recruited children, we first collect skin samples by using a sterile swab to rub skin surface within 25cm^2^ for approximately 20 times back and forth at the piercing site. After disinfection with iodophor, we then collect the residual samples of cerebrospinal fluid (a median of 4ml) and blood (a median of 7 ml) of the children for regular clinical examination, and the remaining CSF and blood after examination are sent to Qingdao oebiotech for 2bRAD-M sequencing. As for the environment samples, we selected needles from the same batch and model as those used in the operation, opened them at the start of the puncture operation, and placed them within the operating tray to expose them to the same environment. Upon completion of the puncture, we gathered the needles used to collect environmental samples with sterile forceps using sterile tubes. All samples were stored in a -80 °C freezer before sequencing.

Our study followed the diagnostic criteria for NBM as outlined in the review “Bacterial meningitis in the neonate: pathogeny, diagnosis and diagnosis” published in the Chinese Journal of Perinatal Medicine, 2016. Specifically, the inclusion criteria were indicated by at least two of the following abnormal clinical observations in CSF: (1) increased white blood cell count (>21x109/L), (2) elevated total protein (>1.0g/L in full-term infants, >1.5g/L in preterm infants), (3) decreased glucose (<1.7mmol/L in full-term infants,<1.1mmol/L in preterm infants), or cerebrospinal fluid bacteria culture positive will be directly diagnosed. After blood and CSF examination, patients with relevant indicators meeting the diagnostic criteria for bacterial meningitis were included in NBM group. Notably, all the enrolled children were patients with elevated blood infection indexes, and clinical manifestations such as fever and poor feeding. These children are at risk for central nervous system infections. The Non-NBM children’s finally diagnosis was sepsis in 6 cases (54.5%), intrauterine infection in 2 cases (18.2%), and pneumonia in 2 cases (18.2%). Moreover, in the NBM children, positive culture results were found in only two cases, which were *Escherichia coli* and non-lactose fermenting *Streptococcus agalactiae*. This further highlights the issue of a low positivity rate associated with traditional culturing methods.

### Ethical approval

In this study, cerebrospinal fluid and blood samples were collected from children after routine clinical examination, which did not increase the burden of additional cerebrospinal fluid and blood collection. This study was approved by the Ethics Committee of Qingdao Women and Children’s Hospital (QFELL-YJ-2022-15). A written statement that formal consent was obtained from the parent/guardian.

### DNA extraction and sequencing

The TIANamp Micro DNA Kit (Tiangen) was used to extract the genomic DNA. 2btags libraries were prepared according to previous studies (Lam et al., 2022; Sun et al., 2022a). First, the genomic DNA was digested with 4 U of BcgI restriction enzyme (NEB). Next, a ligation reaction was performed using a reaction volume of 20 ul containing 10 μl of digested product, 0.2 μM each of library-specific adaptors (Ada1 and Ada2), 1 mM ATP (NEB), 1 × T4 DNA Ligase Buffer, and 800 U T4 DNA ligase (NEB) at 4°C. Then, the BcgI restriction enzyme was inactivated by heating at 65°C. The ligation products were then submitted to PCR amplification in a 40 ul reaction volume consisting of 7 μl ligated DNA, 0.1 μM each primer (Primer1 and Primer2 for Illumina), 0.3 mM dNTP, 1 × Phusion HF buffer, and 0.4 U Phusion high-fidelity DNA polymerase (NEB). The library products were purified with the QIAquick PCR purification kit (Qiagen) and subjected to sequencing via the Illumina HiSeq X™ Ten platform. Library construction and Illumina sequencing were performed at OE BioTech Co., Ltd., Qingdao.

### 2bRAD sequencing data analysis

Raw sequencing reads were demultiplexed and filtered for quality using fastp with default parameters (Chen et al., 2018). Clean reads were obtained from enzyme reads using the following criteria: (1) removing reads with greater than 8% unknown bases; (2) removing reads containing more than 20% of low-quality bases (Q-value ≤ 20). The MAP2B computational pipeline, a machine learning-based tool available on Github (https://github.com/sunzhengCDNM/MAP2B), was used for taxonomic profiling (Sun et al., 2022a; Sun et al., 2022b). It is based on a unique 2bRAD tag database containing species-specific BcgI-derived tags from 48,475 species (including bacteria, fungi, and archaea). First, 2b tags were aligned against a preconstructed unique 2b tag database (BcgI). This initial alignment against a large number of reference genomes in the database generated preliminary profiling results. The output was then passed into a pre-trained classifier to discriminate false positives from true positives. Theoretically existent 2b tags of the true-positive species determined by the classifier were then selected to construct a sample-specific unique 2b-tag database for the second round reads alignment. Finally, the 2b tags were aligned to the sample-specific unique 2b tag database to generate the final taxonomic profiling results (Sun et al., 2021).

### Source tracking

Microbiota source tracking was conducted using the R package FEAST in R 4.0.5 to investigate the invader proximity effect at the community level (Shenhav et al., 2019), the default parameters were used. Specifically, the blood and CSF samples (2bRAD-M profiling results) were used as “sink” separately while the environmental, skin, and negative controls were used as “source” as required by the package FEAST. The results were shown in percentage Bar Charts, with each element indicating the proportion of the microbiota potentially derived from the environments, skin, and sequencing contamination.

### Decontamination (RLD)

To remove contaminated reads in the blood, CSF, and skin 2bRAD-M sequencing data, we developed a novel read filtering method. We consider the reads in the negative control as contaminated reads come from the sample preparation and sequencing due to the high sensitivity of 2bRAD-M for low microbial biomass (Sun et al., 2022a). However, the biomass of the contaminations is not comparable to the blood, CSF, and skin, thus the normalization (subsampling) for the reads in negative control is required when executing the depletion. *D* is defined as the sequences that need to be removed, which are randomly selected from the negative control sequencing reads. It is, calculated as negative control reads number multiplied by the ratio of sample reads to the sum of sample reads and negative control reads (McKnight et al., 2019). We define the reads number in the target sample as T and the reads number in negative control as *N*. *D* can be calculated as:


$$D=\;N*\;\frac{T}{T\;+N}$$

### Statistical analysis

The alpha diversity of microbial composition was measured by the Shannon and species richness indices, calculated using the “Vegan” package. The same package was used to calculate the Bray-Curtis dissimilarity to measure the beta diversity for the multi-omics data. Differences in BC dissimilarity were determined using a non-parametric multivariate analysis of variance (Permanova test) and visualized through principal coordinate analysis (PCoA). The Wilcoxon test was used to determine significance, e.g., the Wilcoxon Rank-sum test was used to compare samples between the two groups using Parallel-Meta 3.5 (Jing et al., 2017).

Here, we used MaAsLin2 (Microbiome Multivariable Associations with Linear Models) to identify multivariable associations between the microbiome and physical indexes (Mallick et al., 2021). MaAsLin2 has been designed to facilitate high-precision association discovery in microbiome epidemiology studies by evaluating and combining the best set of analysis steps. In this study, we employed Total Sum Scaling (TSS) for data normalization and Log transformation (LOG) for data transformation to ensure robust and accurate results. In addition, the correlation between different species and physical indexes was calculated using the Spearman correlation analysis based on the “psych” package. A *p*-value < 0.05 and |r| > 0.6 were considered statistically significant. Data visualization in this study was performed using R, for example, the “ggplot2”, “ggpubr” package were used for boxplot, scatter plot, and line chart, and the “pheatmap” was employed for the heatmaps.

### Random forest modeling

To construct the Random Forest Model, the top-ranking NBM-discriminatory taxa that led to reasonably good fit were identified based on the “rfcv” function in the randomForest package (Teng et al., 2015; Rigatti, 2017). Random Forests models were then trained to classify bacterial meningitis in the training set, which included samples from NBM and non-NBM groups, using CSF, blood, and skin microbial data separately. The results were evaluated using a 10-fold cross-validation approach, and model performance was evaluated using the receiver operating characteristic (ROC) curve. Default parameters of the Random Forest were applied (ntree = 5,000, using the default mtry of p/3, where p is the number of input taxa) (Sun et al., 2022c).

## Results

### Experimental design and participants

To investigate the prevalence and characteristics of CNS infections in neonates admitted to the NICU of Qingdao Women and Children’s Hospital. A total of 23 neonates were enrolled in the study between January 1, 2021 and March 1, 2022, and suspected of having CNS infectious diseases. Based on their cerebrospinal fluid (CSF) examination results, the patients were divided into two groups: those with neonatal bacterial meningitis (NBM, n=12) and those without CNS infection (non-NBM, n=11).

Our study collected a range of clinical data in addition to the diagnosis. This included demographic information such as gestation days, neonates’ weight, gender, and age (days after birth), as well as other clinical details such as antibiotic and probiotic usage and feeding formula (as shown in **Table 1**). All infants included in the study clinically recovered and were discharged from the hospital after receiving treatment. A total of 66 samples were collected from the CSF, blood, and skin of neonates, including 12 CSF samples, 11 blood samples, and 10 skin samples in the NBM group; and 11 CSF samples, 11 blood samples, and 11 skin samples in the non-NBM group.

**Table 1:** Mean value of physiological and biochemical test results of the infant participants.

Category		Index (IQR)	NBM (N = 12)	non-NBM (N = 11)	*P*-value^*^
**General** **Index**		Mother Age	31 (29-33)	31 (28-34)	0.708
		Gestational Age	39 (34-43)	40 (38-42)	0.776
		Infant Age (Week)	3 (1.6-3.4)	1 (0.2-1.6)	0.072
		Infant Weight	2.9 (2.0-3.6)	3 (2.4-3.6)	0.815
		PROM (%)	3 (25.0%)	4 (36.4%)	0.554
		Antibiotics (%)	4 (33.3%)	6 (54.5%)	0.305
		Delivery mode (%)	4 (33.3%)	2 (18.2%)	0.409
**Blood** **Index**	**Blood** **Routine** **Examination**	WBC	13.3 (9.6-14.0)	16.6 (12.0-21.2)	0.241
		Hb	134.5 (121.5-150.8)	150.7 (136.5-171.5)	0.248
		PLT	190.8 (74-341)	261 (221.5-298.5)	0.225
		NEUT%	50 (46.6-58)	58 (44.0-73.9)	0.375
		MONO%	10 (10.1-12.8)	7.6 (6.0-10.8)	0.217
		LYM%	29.9 (28.3-37.2)	27.9 (16.5-47.0)	0.778
		CRP	61.6 (8.0-118.8)	29.4 (0.8-19.8)	0.267
		PCT	23.1 (1.5-33.2)	16.1 (1.3-18.0)	0.577
	**Blood** **Metabolism**	Albumin	32.5 (28.0-36.5)	31.4 (33.3-37.0)	0.751
		LDH	459 (292.2-425.3)	549.4 (451.4-857.3)	0.650
**CSF** **Index**	**CSF Routine** **Examination**	CSF WBC	3559.2 (56.5-5725.5)	8.1 (1.5-13.5)	0.015
		Pandy (%)	9 (75.0%)	9 (81.8%)	0.948
	**CSF** **Metabolism**	CSF Protein	205.9 (123.4-273.7)	121.4 (84.8-162.7)	0.042
		CSF Glu	2.5 (1.2-2.6)	3.4 (2.9-3.7)	0.197

^*^ All continuous numerical variables were analyzed using Student’s t-test to determine their statistical significance, and nominal categorical variables (PROM, and Antibiotics) were compared using the chi-square test between the NBM and non-NBM groups.

The mothers of the recruited infants are ethnically identical (Asian). Comparison of the mothers’ age, gestational age and delivery mode between the NBM and non-NBM revealed no significant differences (*p* values of 0.708, 0.776 and 0.409 respectively). Then, we compared routine physiological indicators of the neonates, such as birth weight, Premature rupture of membranes (PROM), and antibiotic usage (if any), and found no differences (*p* values of 0.072, 0.815, 0.554, and 0.305 respectively). Moreover, we further compared the biochemical test indicators in blood and CSF between the two groups and identified no significant differences in blood regarding the White Blood Cell (WBC), Hemoglobin (Hb), Platelet count (PLT), Neutrophil proportion (NEUT%), Monocyte proportion (MONO%), Lymphocyte proportion (LYM%), C-reactive protein (CRP), Procalcitonin (PCT), Albumin, Lactate dehydrogenase (LDH), and neither the level of Pandy and Glucose in CSF (*p* > 0.05). As previously mentioned, the CSF WBC and CSF total protein levels are significantly higher in the NBM group than non-NBM group (*p* values of 0.015 and 0.042 respectively).

### Unique microbiota associated with blood and cerebrospinal fluid

**Figure 1:**
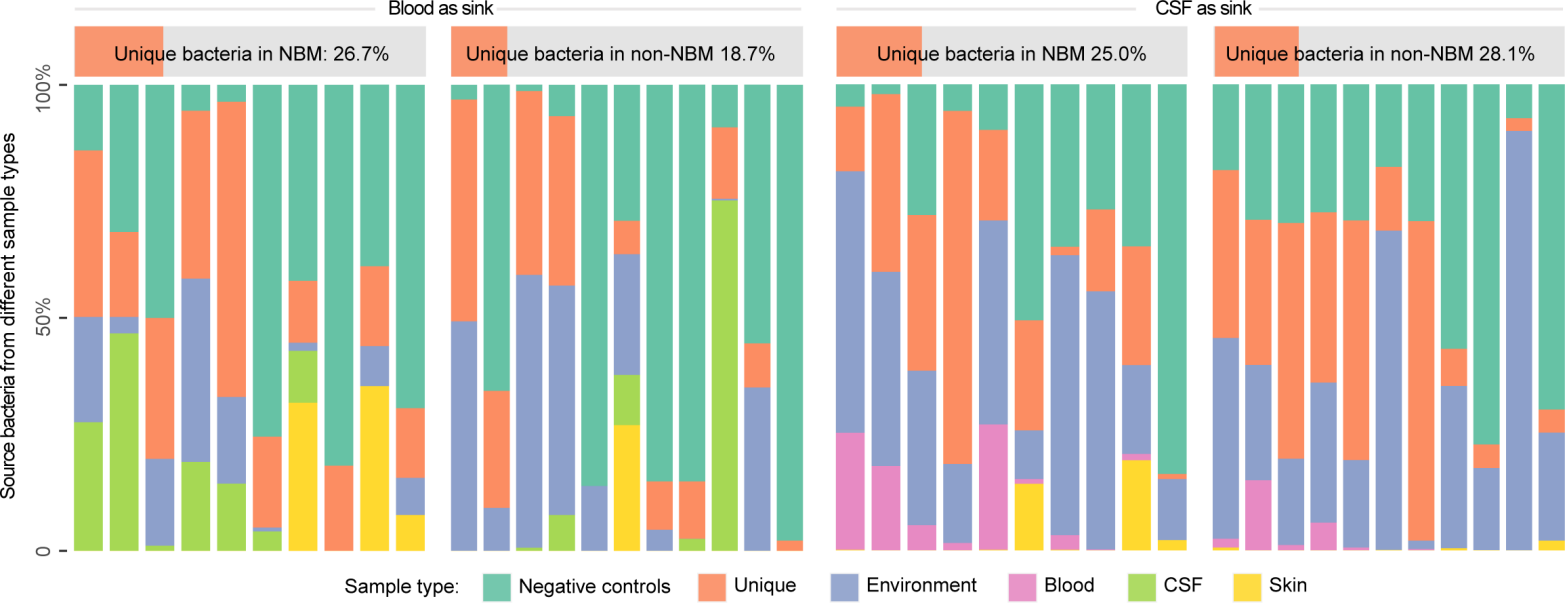
Source tracking for the blood and CSF microbiota before decontamination. We performed source tracking to explore the blood and CSF tissue-specific microbiota (the proportion that cannot be explained by the source tracking). NBM and non-NBM were separately illustrated within different tissue types. Based on the source tracking results, the unique microbiota belonging to the blood tissue is average of 26.7% and 18.7% in the two groups, while the unique microbiota only existed in the CSF tissue is 25.0% and 28.1% separately, indicating the existence of blood and CSF tissue specific taxa in both the NBM and non-NBM group.

Both blood and CSF were traditionally considered to be sterile environments. However, with the development of metagenomic approaches, blood-based and CSF-based microbiota attracted great attention. To explore whether there are any blood or CSF tissue-specific taxa, we first performed source tracking to trace the possible source of the taxa in the blood and CSF microbial profiling results by searching against environments (including the surface of skin and sampling instruments) and negative controls in sequencing (**Figure 1**). In particular, each of the blood or CSF samples were considered as sink while the environments and negative controls were treated as source in the source tracking analysis (Methods). We found that: (1) when using blood samples as sink, there is an average of 26.7% and 18.7% in the NBM and non-NBM groups that cannot be explained by the environments and negative controls, indicating the existence of the unique microbiota belonging to the blood tissue; (2) when using CSF samples as sink, the unique microbiota only existed in the CSF tissue were identified to be 25.0% and 28.1% separately, suggesting the existence of CSF specific taxa in both the NBM and non-NBM group as well.

### Microbial diversity before and after decontamination

Although the source tracking results revealed the existence of blood and CSF-specific taxa, there is an obvious overlapped proportion between blood/CSF and negative controls, which may impact downstream analysis e.g., leading to false positives in differential analysis. We then sought to deplete such noise information from blood and CSF samples by a novel decontamination method using negative controls (**Figure S1**, Methods). Specifically, we compared the short reads (2b tags) in the blood, CSF, and skin samples with the short reads in negative controls, and then discarded those overlapped reads before profiling process (Methods). In addition, we also employed a conventional decontamination method in which we set up a relatively strict abundance threshold (0.01%) and used a potential contaminant taxa as a reference (Salter et al., 2014) to remove contaminations. To illustrate such decontamination is effective, we compared the microbial diversity before and after reads depletion.

**Figure 2:**
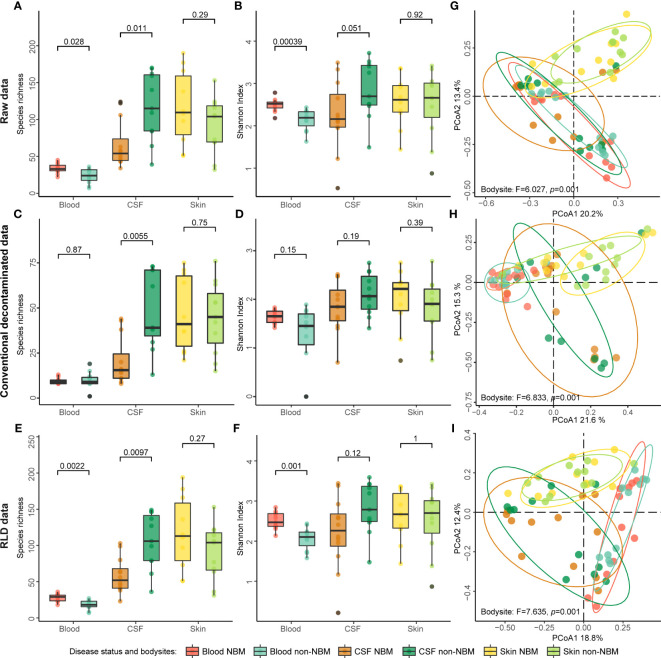
Alpha and beta diversity of the raw and decontaminated 2bRAD sequencing data. **(A–F)** Species richness and Shannon index of the raw data and decontaminated data were calculated separately to evaluate the alpha diversity of NBM and non-NBM individuals from different body sites (blood, CSF, and skin). **(G–I)** Beta diversity was implemented using the Bray-Curtis distance-based PCoA. Both species richness and Shannon index showed that this method of contamination removal did not significantly impact the comparisons of the alpha diversity of NBM and non-NBM individuals (*p* > 0.05).

Previous studies have shown that higher alpha diversity in the microbiome is associated with healthy status of host (Armour et al., 2019; Astbury et al., 2020; Manor et al., 2020). To investigate whether there are differences in alpha diversity between NBM and non-NBM groups, we calculated the species richness and the Shannon index of the microbiomes in blood, CSF, and skin samples. We found both the species richness and Shannon index were consistent in the comparison for NBM and non-NBM groups regardless of whether raw or decontaminated data were used: (1) For blood samples, the NBM group had significantly higher species richness and Shannon index values than the non-NBM group (**Figures 2A–F**, *p*< 0.05); (2) However, for CSF samples, an opposite comparison results were found, with the NBM group having lower alpha diversity than the non-NBM group (**Figures 2A–F**, species richness: *p*< 0.05, Shannon index: *p* > 0.05); (3) And there were no significant differences in alpha diversity between the NBM and non-NBM group in skin samples (**Figures 2A–F**, *p* > 0.05). This indicates that NBM patients are characterized with higher microbial alpha diversity in CSF but lower microbial alpha diversity in the blood compared to relatively healthy neonates.

As for the beta diversity, we found the discriminating power of the microbiota for NBM is enlarged after decontamination, e.g., the separation trend of microorganisms from different body sites was more significant (**Figures 2G–I**, Permanova test, Raw data: F = 6.027, *p* = 0.001; Conventional decontamination method: F = 6.833, *p* = 0.001; RLD: F = 7.635, *p* = 0.001). However, there was no obvious separation in the taxonomic structure of NBM and non-NBM groups in blood, CSF, and skin (**Figure S2**, Permanova test, Raw data: F = 0.728, 1.196, and 0.830, *p* = 0.491, 0.272, and 0.664; Conventional decontamination method: F = 2.447, 0.981, and 1.234, *p* = 0.021, 0.413, and 0.237; RLD: F = 1.117, 1.015, and 0.846, *p* = 0.336, 0.354, and 0.652);. In addition, here we see RLD outperformed the conventional decontamination method in differentiating microbiota from different body sites. All the above suggest that the decontamination method used in this study can effectively correct beta diversity analysis results without affecting alpha diversity analysis.

**Figure 3:**
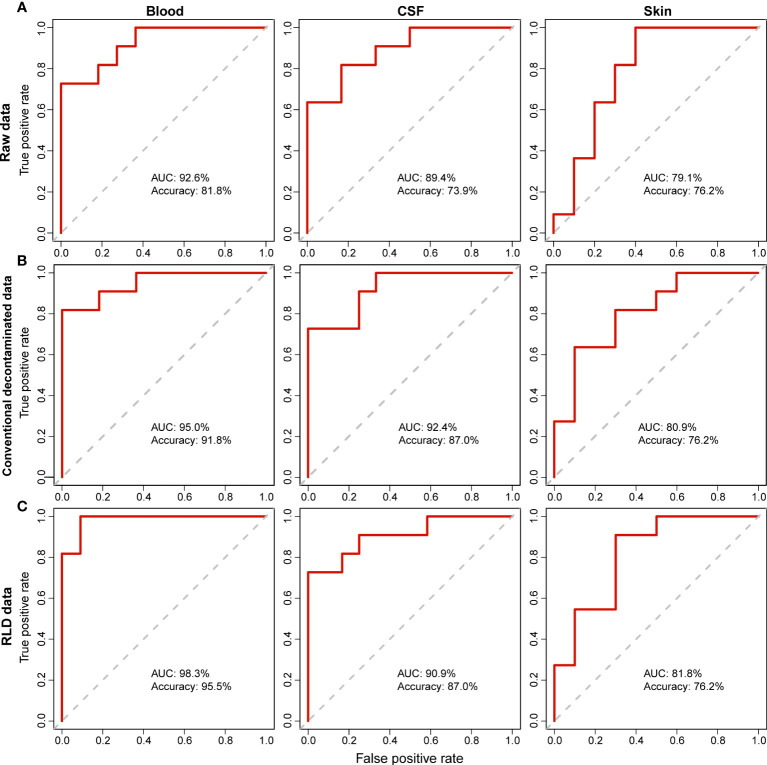
Comparison of the Random Forest (RF) models using the raw and decontaminated data in determining NBM. For both **(A)** raw data **(B)** decontamination data by conventional method, and **(C)** decontamination data by RLD, RF prediction models were built using the relative abundance of taxa identified in different body sites (blood, CSF and skin) to diagnose the infection (NBM). The robustness of the RF models was evaluated by the Area Under the Receiver Operating Characteristic Curve (AUC).

To further demonstrate the rationality and effectiveness of decontamination, we trained Random Forest (RF) models to diagnose NBM from non-NBM neonates. The robustness of the RF models was evaluated by the Area Under the Receiver Operating Characteristic Curve (AUC). We then found that, RF models based on raw data (**Figure 3A**, Raw data: Accuracy is 81.8%, 73.9%, and 76.2% respectively; AUC is 92.6%, 89.4%, and 79.1% respectively) can be improved after contamination removal. The prediction accuracy of the refined RF models established from blood, CSF and skin samples are: (1) the conventional method: Accuracy is 91.8%, 87.0%, and 76.2% respectively; AUC is 95.0%, 92.4%, and 80.9% respectively (**Figure 3B**); (2) RLD: Accuracy is 95.5%, 87.0%, and 76.2% respectively; AUC is 98.3%, 90.9%, and 81.8% respectively (**Figure 3C**). This also suggests a good performance and necessity of the decontamination in correcting the noise introduced by sequencing.

### Microbial signatures of NBM form blood, cerebrospinal fluid, and skin

**Figure 4:**
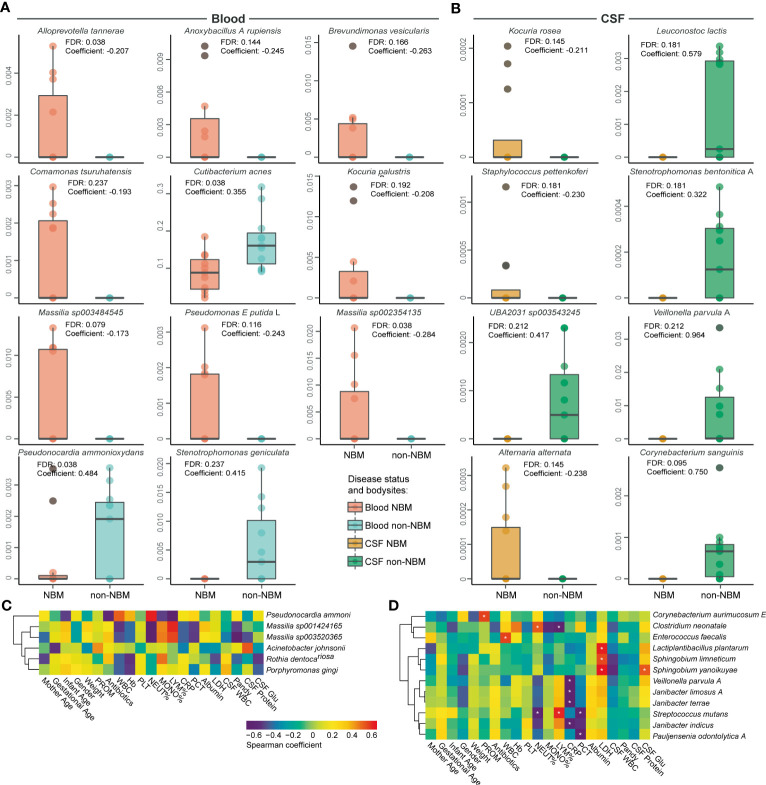
Differential and associated species in NBM. Boxplots of the differentially abundant species between the NBM and non-NBM groups in **(A)** blood and **(B)** CSF samples, no significant difference between the two groups were found in skin samples. **(C, D)** Correlations between differential species and physiological indicators in **(C)** blood and **(D)** CSF samples (defined by a Spearman coefficient of *p*< 0.05, and |r| > 0.6). FDR<0.2 were marked with an asterisk.

After decontamination, we next sought to explore the microbial biomarkers that can distinguish NBM from non-NBM samples. To perform the differential analysis and rule out the influence from the confounders (such as body site, antibiotics, septicemia, weight, etc.), we employed MaAsLin2 to identify the biomarkers for NBM. We found that: (1) In blood (**Figure 4A**), *Alloprevotella tannerae*, *Anoxybacillus A rupiensis*, *Brevundimonas vesicularis*, *Comamonas tsuruhatensis*, *Kocuria palustris*, *Massilia sp003484545*, *Pseudomonas E putida*, and *Massilia sp002354135* were significantly higher in the NBM group, while *Cutibacterium acnes* was significantly higher in the non-NBM group; (2) In CSF (**Figure 4B**), *Kocuria rosea*, *Staphylococcus pettenkoferi*, and *Alternaria alternata* were significantly higher in the NBM group compared to the non-NBM group, while *Leuconostoc lactis*, *Stenotrophomonas bentonitica A*, *UBA2031 sp003543245*, *Veillonella parvula A* and *Corynebacterium sanguinis* were significantly higher in the NBM relative to the non-NBM group; (3) There were no significant differences in skin samples between the NBM and non-NBM groups.

In order to further investigate the correlation between differential species and physiological indicators, a spearman correlation analysis was conducted. We found that in blood samples (**Figure 4C**), the relative abundance of *Pseudonocardia ammonioxydans*, *Massilia sp001424165*, *Massilia sp003520365*, *Acinetobacter johnsonii*, and *Rothia dentocariosa* significantly associated with blood and CSF physiological indicators. As an important indicator of disease state, an increase in the relative abundance of *Acinetobacter johnsonii* in CSF samples significantly correlated with the increase of patient’s CSF protein level (r > 0.6, *p*< 0.05). In CSF samples (**Figure 4D**), *Corynebacterium aurimucosum E*, *Clostridium neonatale*, *Enterococcus faecalis*, *Lactiplantibacillus plantarum*, *Sphingobium limneticum*, *Sphingobium yanoikuyae*, *Veillonella parvula A*, *Janibacter limosus A*, *Janibacter terrae*, *Streptococcus mutans*, *Janibacter indicus*, and *Pauljensenia odontolytica A* were found to be associated with disease-related physiological indicators. Moreover, the increase of CSF WBC in the NBM group were found to be negatively associated with the abundance of *Clostridium neonatale*, *Sphingobium limneticum*, and *Sphingobium yanoikuyae* (r< 0.6, *p<* 0.05). In summary, we identified 11 and 8 potential biomarkers for NBM in blood and CSF separately and discovered 16 and 35 microbial species that highly correlated with the physiological indicators in blood and CSF.

## Discussion

Our study aims to improve the diagnostic accuracy of neonatal bacterial meningitis by using 2bRAD-M sequencing that has been proven to be effective in dealing with low microbial biomass and highly degraded DNA samples. Additionally, we developed a new decontamination method that can remove potentially contaminated DNA to enhance the reliability of the microbial profiles. As a result, the difference in the body sites and discriminating power of the blood and CSF microbiota for NBM was improved after decontamination. Overall, our study contributes to identifying the risk factors in the microenvironments associated with CNS infections in neonates and provides a glimpse into the early diagnosis and treatment of neonatal bacterial meningitis in the NICU.

One of the main findings of this paper is the identification of distinct microbial signatures in the blood and CSF of patients with NBM. Specifically, in the blood, we observed significant variations in the abundance of 11 bacterial species.: *Massilia sp003484545*, *Massilia sp002354135*, *Pseudomonas E putida*, *Alloprevotella tannerae*, *Anoxybacillus A rupiensis*, *Brevundimonas vesicularis*, *Kocuria palustris* and *Comamonas tsuruhatensis* were found to be more abundant in the NBM group. In contrast, *Cutibacterium acnes*, *Pseudonocardia ammonioxydans* and *Stenotrophomonas geniculata* were found to have a higher abundance in uninfected individuals’ blood (non-NBM group). Some of them have been reported as pathogenic bacteria, e.g., *P. putida* can cause various infections in newborns (Baykal et al., 2022), and *Comamonas testosterone* (previously known as *Pseudomonas testosterone*) is a common pathogen in its genus. However, many of these species, including *Anoxybacillus rupiensis*, *Brevundimonas vesicularis*, *Pseudonocardia ammonioxydans*, and *Kocuria palustris* have rarely been reported as pathogens and are commonly found in soil, rivers, hot springs, and marine organisms (Derekova et al., 2007; Popowska et al., 2010; Martin et al., 2013). Additionally, some human commensal bacteria like *Cutibacterium acnes* and *Stenotrophomonas geniculata* were also identified, although there is limited research on their medical significance.

In the CSF, *Corynebacterium sanguinis*, *Leuconostoc lactis*, *Stenotrophomonas bentonitica A*, *Veillonella parvula A* and *UBA2031 sp003543245* were found to have higher abundance in non-NBM group, while *Alternaria alternata*, *Kocuria rosea* and *Staphylococcus pettenkoferi* were found to have a higher abundance in NBM group. Notably, *Alternaria alternata* is a species of ascomycete fungi within the Pleosporaceae family, which is associated with allergic asthma or allergic pneumonia (Stern et al., 2008; Loghmani et al., 2017) and has been identified as an infectious agent in cases of mycotic keratitis, particularly in association with soft contact lenses and corneal transplantation (Huerva and Soldevila, 2017). *Kocuria rosea* and *Staphylococcus pettenkoferi* can also be an opportunistic pathogen (Ahmad-Mansour et al., 2021), causing infections in immunocompromised patients (Villafañe et al., 2012). It has been reported to cause multiple site infections in recent years, such as endocarditis (Srinivasa et al., 2013; Moreira et al., 2015), meningitis (Sipahi et al., 2014), bacteremia (Nudelman et al., 2022), and canaliculitis (Ali et al., 2014).

Contaminations from the environment and host is currently one of the most concerning and challenging issues in the microbiome study of low-biomass samples. We believe that to solve this issue, it requires highly sensitive metagenomic sequencing methods like 2bRAD-M, complemented with appropriate decontamination methods. We acknowledge the importance of validating metagenome sequencing results with standard laboratory tests such as culture and PCR. Therefore, we performed qPCR validation of the microbes identified by 2bRAD-M in this study. However, due to the limitations of unavailable primers, we were only able to verify the presence of several bacteria. For instance, we detected *Cutibacterium acnes*, *Escherichia coli*, and *Enterococcus faecalis* in the CSF. Additionally, in the blood, we detected not only the above three bacteria but also *Klebsiella pneumoniae*, thus providing strong evidence for the accuracy of 2bRAD-M in detecting blood and CSF microbiomes.

Regarding decontamination methods, we compared a conventional method (using a strict threshold and potential decontaminant taxa as a reference) with our method (RLD), and we found that both methods performed better in determining the microbiome from different body sites and disease status, with RLD slightly outperforming the conventional method. In addition, there are currently many useful tools for eliminating contamination introduced in the wet lab using negative and positive controls (Gloor et al., 2017; Morton et al., 2019; Lin and Peddada, 2020; Austin et al., 2023). However, these tools process from the taxonomic profiles without handling the sequencing data. The method we used in this study innovatively eliminates contamination from the more fundamental data, which is at the reads level, and may be a more friendly decontamination method for low microbial biomass sequencing. In future work, we will further compare our method with others to test this hypothesis.

There are still some limitations regarding the sample size. Thus, in future work, we plan to validate the identified disease-associated biomarkers through a larger cohort with qPCR and digital PCR, and incorporate metabolomic data to better understand the pathogenesis of NBM. In summary, our study identified NBM biomarkers from multiple body sites through the 2bRAD-M sequencing method and an innovative decontamination method, pioneering in providing microbial signatures for the study of the occurrence, prevention, and diagnosis of NBM.

## Data availability statement

The datasets presented in this study can be found in online repositories. The names of the repository/repositories and accession number(s) can be found below: https://ngdc.cncb.ac.cn/search/?dbId=gsa&q=CRA009684, CRA009684.

## Ethics statement

The requirement of ethical approval was waived by the Ethics Committee of Qingdao Women and Children’s Hospital for the studies involving humans because We did not waive the ethical approval. The studies were conducted in accordance with the local legislation and institutional requirements. Written informed consent for participation was not required from the participants or the participants’ legal guardians/next of kin because We filled out a written informed consent form. Written informed consent was obtained from the individual(s), and minor(s)’ legal guardian/next of kin, for the publication of any potentially identifiable images or data included in this article.

## Author contributions

The authors confirm contribution to the paper as follows: study conception and design: ZS and XL. data collection: YH. Analysis and interpretation of results: MZ and JL. Draft manuscript preparation: YH, ZS, and MZ. All authors contributed to the article and approved the submitted version.

## Supplementary material

Supplementary Figure 1 The workflow of decontamination for 2bRAD sequencing data. We first compare the sequencing reads in the negative control with the target sample, and then remove the overlapped reads from the latter based on a corrected reads number (see **Methods**) to avoid the bias introduced by the unbalanced sequencing depth of the target sample and negative control. Then the clean sequencing data will be passed to the profiling process for downstream analysis. The decontamination process is separately conducted for each of the target samples.

Supplementary Figure 2 Comparison of the raw and decontaminated sequencing data in beta diversity analysis. A PCoA analysis was conducted using the Bray-Curtis distance metric to assess the clustering patterns of NBM and non-NBM individuals in blood, CSF, and skin samples, using both raw and decontaminated data.
