# Supplementary figures and images for: Microbial signatures of neonatal bacterial meningitis from multiple body sites
Source: Front Cell Infect Microbiol. 2023 Aug 22;13:1169101. doi: 10.3389/fcimb.2023.1169101 (PMC10477713; doi:10.3389/fcimb.2023.1169101)

Figure S1

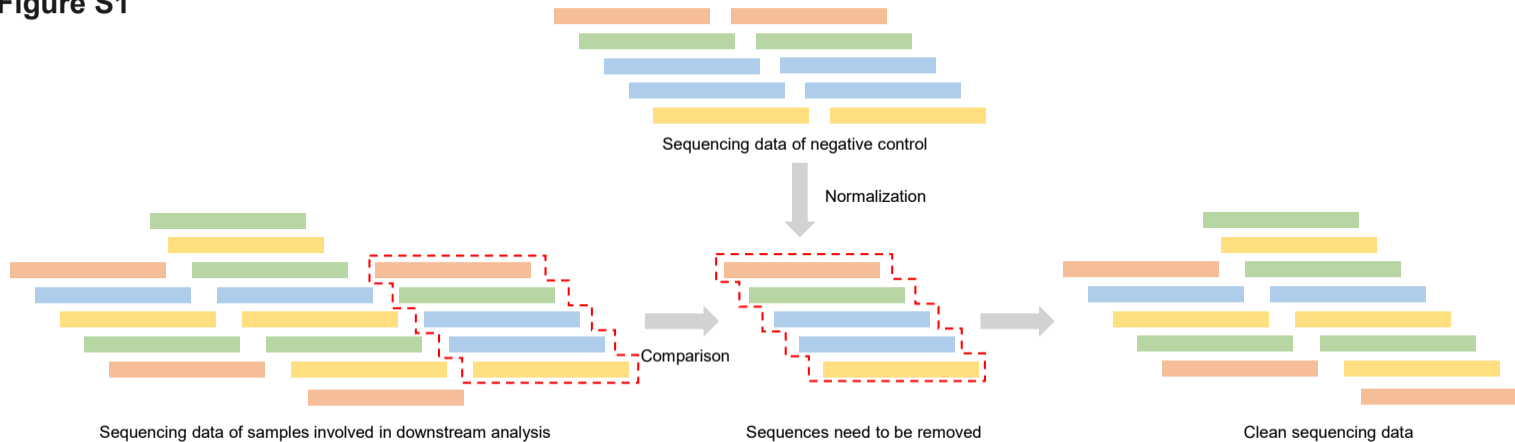

Supplement: Supplementary Figure 1 — The workflow of decontamination for 2bRAD sequencing data. We first compare the sequencing reads in the negative control with the target sample, and then remove the overlapped reads from the latter based on a corrected reads number (see Methods) to avoid the bias introduced by the unbalanced sequencing depth of the target sample and negative control. Then the clean sequencing data will be passed to the profiling process for downstream analysis. The decontamination process is separately conducted for each of the target samples. [file Image_1.pdf]

Figure S2

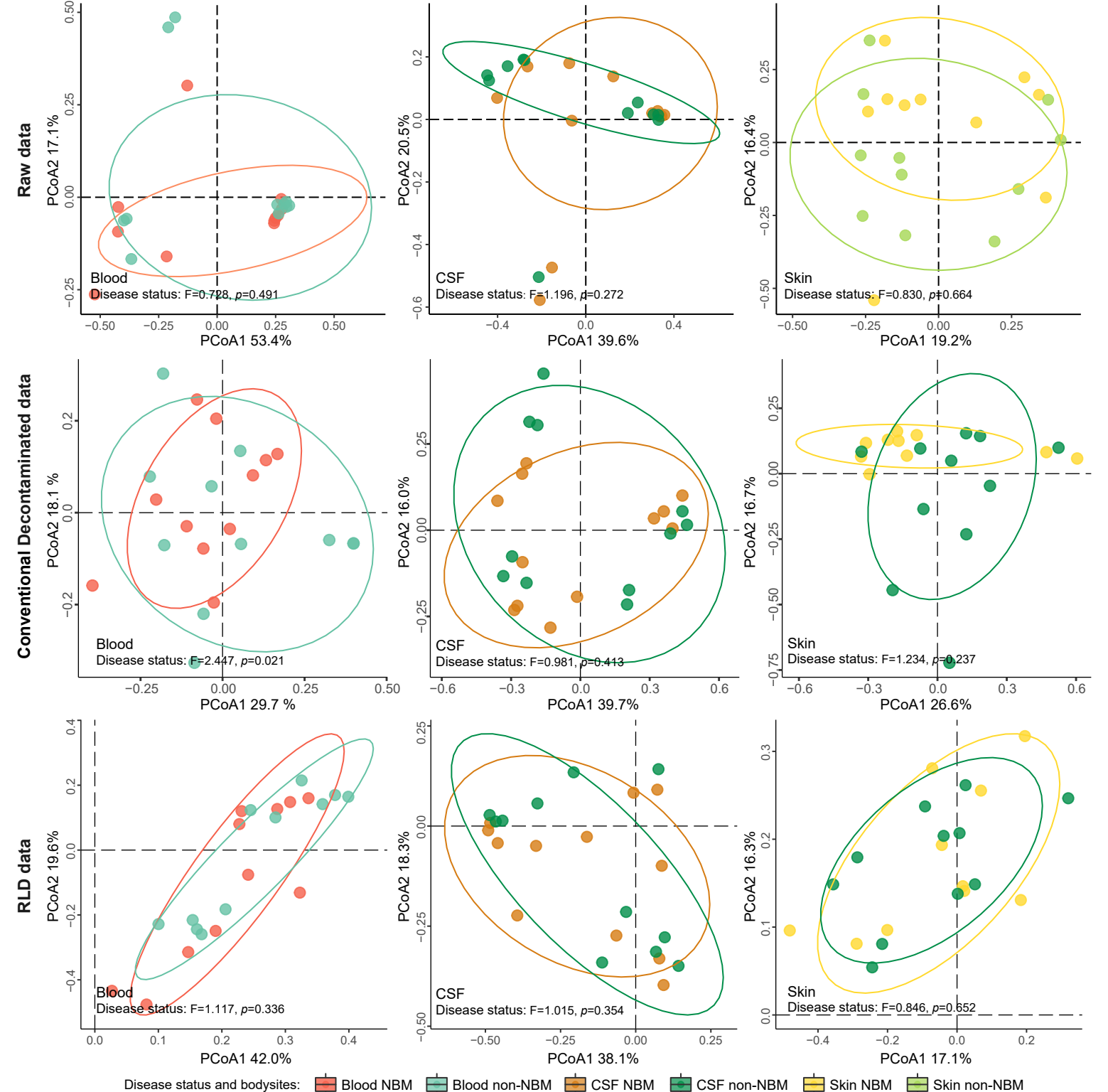

Supplement: Supplementary Figure 2 — Comparison of the raw and decontaminated sequencing data in beta diversity analysis. A PCoA analysis was conducted using the Bray-Curtis distance metric to assess the clustering patterns of NBM and non-NBM individuals in blood, CSF, and skin samples, using both raw and decontaminated data. [file Image_2.pdf]
